# Network pharmacology and *in vitro* testing of *Theobroma cacao* extract’s antioxidative activity and its effects on cancer cell survival

**DOI:** 10.1371/journal.pone.0259757

**Published:** 2022-04-14

**Authors:** Priyanka P. Patil, Vishal S. Patil, Pukar Khanal, Harish R. Darasaguppe, Rajitha Charla, Arati Bhatkande, Basanagouda M. Patil, Subarna Roy

**Affiliations:** 1 KLE College of Pharmacy Belagavi, KLE Academy of Higher Education and Research (KAHER), Belagavi, Karnataka, India; 2 Indian Council of Medical Research- National Institute of Traditional Medicine, Belagavi, Karnataka, India; 3 Department of Pharmacology, NGSM Institute of Pharmaceutical Sciences (NGSMIPS), Nitte (Deemed to be University), Mangalore, India; Institute for Biological Research, University of Belgrade, SERBIA

## Abstract

*Theobroma cacao* L. is a commercially important food/beverage and is used as traditional medicine worldwide against a variety of ailments. In the present study, computational biology approaches were implemented to elucidate the possible role of cocoa in cancer therapy. Bioactives of cocoa were retrieved from the PubChem database and queried for targets involved in cancer pathogenesis using BindingDB (similarity index ≥0.7). Later, the protein-protein interactions network was investigated using STRING and compound-protein *via* Cytoscape. In addition, intermolecular interactions were investigated *via* molecular docking. Also, the stability of the representative complex Hirsutrin-epidermal growth factor receptor (EGFR) complex was explored using molecular dynamics simulations. Crude extract metabolite profile was carried out by LC-MS. Further, anti-oxidant and cytotoxicity studies were performed in Chinese hamster ovary (normal) and Ehrlich ascites carcinoma (cancer) cell lines. Herein, the gene set enrichment and network analysis revealed 34 bioactives in cocoa targeting 50 proteins regulating 21 pathways involved in cancer and oxidative stress in humans. EGFR scored the highest edge count amongst 50 targets modulating 21 key pathways. Hence, it was selected as a promising anticancer target in this study. Structural refinement of EGFR was performed *via* all-atom molecular dynamics simulations in explicit solvent. A complex EGFR-Hirsutrin showed the least binding energy (-7.2 kcal/mol) and conserved non-bonded contacts with binding pocket residues. A stable complex formation of EGFR-Hirsutrin was observed during 100 ns MD simulation. *In vitro* studies corroborated antioxidant activity for cocoa extract and showed a significantly higher cytotoxic effect on cancer cells compared to normal cells. Our study virtually predicts anti-cancer activity for cocoa affected by hirsutrin inhibiting EGFR. Further wet-lab studies are needed to establish cocoa extract against cancer and oxidative stress.

## Introduction

Chemotherapeutic agents are extensively used in the management of cancer pathogenesis and metastasis. However, very often, the normal cells also suffer collateral damage due to chemotherapy as there is no selective interaction of the chemotherapeutic agents against normal and malignant cells. Further, maintenance of balance between wanted and unwanted effects has been a serious challenge during chemotherapy [1]. Among the known causes of cancer, the production of reactive oxygen species (ROS), and the development of protection against antioxidants in cells are well documented. Under conditions of oxidative stress, overproduction of ROS leads to necrobiosis *via* apoptosis or necrosis. Also, cancer cells extensively exhibit glycolysis to produce energy which is required for neoplastic cell proliferation and disruption of glycolysis has been suggested as a possible strategy for cancer therapy [2]. Since early 19^th^ Century, prevention of certain cancers have been suggested by adoption of proper nourishment and it has been postulated that nutrition might impact the risk of cancer [3]. There is a growing list of herbal medicines and botanicals that have been reported to possess anticancer activities as well [4]. However, the inadequacy of sufficient scientific data on these botanicals to manage complex polygenic pathogenesis like cancer, coupled with lack of information on the number and concentrations of active phytoconstituents, their molecular roles in the disease pathways have been limited in practice.

Cocoa derived from the plant *Theobroma cacao* L. (family Malvaceae) renders tremendous health benefits including cardioprotective [5], anti-cancer [6], anti-inflammatory [7], anti-diabetic [8], anti-obesity [9], and wound healing properties [10]. Further, it has been reported to reduce blood pressure [11], asthma complications [12], and improve cognitive function [13]. Since cocoa is the primary ingredient of chocolates and drinks, they are composed of the highest levels of flavonoids amongst the commonly consumed foods. Interestingly, these have been traditionally used as medicines to treat inflammation, pain, and numerous other diseases [14]. Additionally, cocoa contains phenolic bioactives that may act as checkpoints in cancer prevention/progression and flavonoids (catechin, epicatechin, and procyanidins) that exhibit the antioxidant activity and alter the immune response of cytokines, inflammatory response, cellular proliferation, and cell adhesion as reported from *in vitro* and *ex vivo* experiments [15–17]. The antioxidant activity of phenolic compounds has redox properties to act as reducing agents, ROS scavengers, hydrogen bond donors, and metal ions chelators [14]. Despite the availability of such information on the phytoconstituents of cocoa, to the best of our knowledge, the possible mechanism of action of cocoa in management of cancer has not been reported yet.

System biology components including network pharmacology describe the complex relationships between biological systems, drugs, and disease [18]. It also elucidates the possible mechanisms of action of complex bioactive substances through analyzing large amounts of data and identifying synergistic effects in multiple pathogeneses. Further, target-based network pharmacology is a promising approach for drug discovery and development of next-generation herbal or herbal formulations [18]. Hence, in the present study, an attempt was made to identify the probable potential protein targets and molecular pathways modulated by bioactives of cocoa against oxidative stress and cancer using the network and reverse pharmacology approaches and to study the basic antioxidant and anticancer activities through *in vitro* assays.

## Materials and methods

### *In silico* pharmacology

#### Mining of bioactives and their target prediction

The bioactives from cocoa were listed from the literature and their structures in “smile” and “sdf” file format were then retrieved using publicly available small molecule databases like phytochemical interactions database (PCIDB; *https://www.genome.jp/db/pcidb/*), Dr. Dukes DB) (*https://phytochem.nal.usda.gov/phytochem/search*). The targets were predicted using BindingDB correspondence to the known ligand molecules having minimum similarity of >0.7 and their Gene IDs were retrieved from UniProtKB (*https://www.uniprot.org*) database.

#### Pathway and network construction

A set of Gene IDs was submitted to search tool for the Retrieval of Interacting Genes/Proteins (STRING; *https://string-db.org/*) [19] 11.0v to identify the protein-protein interaction and pathways modulated by the predicted targets. The overall pathways modulated by the gene set were identified using the KEGG pathway (https://www.genome.jp/kegg/). The network between compounds, targets, and pathways were constructed using Cytoscape [20] *ver* 3.6.1. Biological interactions among them were interpreted based on edge count. The map node size was set from ’low values to small sizes’ and the map node color from ’’low value to bright colors" was set for the network [21,22].

#### Epidermal Growth Factor Receptor (EGFR) structure refinement and active sites assessment

EGFR is a potential cancer target and a highly connected target within the network was selected to identify phytochemical binding affinity and their interactions with its active site residues. EGFR (PDB: 6LUB) x-ray crystallographic structure was chosen from the RCSB PDB database (*https://www.rcsb.org/*), visualized for its missing amino acid, and remodeled by homology modeling approach with Uniport ID: P00533 as a query sequence [23]. Total 100 structures were generated of which structure with the least DOPE score and having least RMSD value was chosen for further structural refinement using MD simulations.

#### Least potential energy (PE) conformation by molecular dynamics (MD) simulation

We used Desmond molecular modeling software version 6.1 [24] for MD simulations. All-atom explicit MD simulation for 50 ns was performed with the OPLS force field. The modeled EGFR structure was solvated using a simple point charge (SPC) water model in the cuboidal box (10Å × 10Å × 10Å) periodic boundary condition. The system was neutralized by adding six positively charged counterions (Na). Further, to restrain the geometry of water molecules, bond angles, and bond lengths of heavy atoms, the SHAKE algorithm was applied and the Particle Mesh Ewald (PME) method was used to treat long-range interactions. The Lennard-Jones interactions cut-off was set to 10Å. The system was then subjected for production MD run followed by energy minimization using default parameters *via* pressure (1.01325 bar), and temperature (300 K). The trajectory was analyzed to check the structural stability and to obtain the lowest PE confirmation of the EGFR.

#### Molecular docking

Three-dimension structures of each bioactive and known EGFR inhibitor Erlotinib were retrieved from the PubChem database in “sdf” file format and converted into “PDB” file format using Biovia Discovery Studio Visualizer 2019. All the small molecules were subjected for energy minimization using the “mmff94” force field in Open babel and the least energy conformation was chosen for docking. The least potential energy conformation of EGFR was extracted from the trajectory and selected for the docking study. Docking was performed using AutoDock vina via executed through POAP pipeline [25].The grid was set around active site residues with box dimensions box center x = 2.8125, y = -9.6422, z = -0.175; and box size 26Å in all directions with spacing 1Å. The exhaustiveness was set to 100. Docking results were analyzed using a discovery studio visualizer to infer the intermolecular interaction of bioactives with the EGFR target.

#### Protein-ligand complex stability

The docked complexes with the least binding energy and maximum interaction with active site residue were subjected to 100ns MD simulation using similar parameters used for EGFR MD. Total three replicas of MD simulation were run to get plausible data from the study using the same starting structure and parameters. Trajectories generated were analyzed to investigate stability and intermolecular interactions.

#### Drug-likeness and side effects prediction

The MolSoft web server (http://www.molsoft.com) was used to predict drug-likeness of selected bioactives and Erlotinib. Similarly, ADVER-Pred [26] an online server was used to predict the possible side effects of bioactives and Erlotinib.

### Experimental pharmacology

#### Plant material

Cocoa pods were obtained from Sirsi (14°.34’38.7984 N, 74°.58’21.288 E), Uttar Kannada District, Karnataka, India, identified and certified by a qualified plant taxonomist at ICMR- NITM, Belagavi. The voucher specimen of the same has been deposited in ICMR-NITM with accession number RMRC-1392 for future reference.

#### Extraction

The dried cocoa beans were crushed and defatted using ten folds of petroleum ether to remove the fat using the Soxhlet apparatus. Defatted and dried powder of cocoa was then extracted through cold maceration technique with ethanol: water (80%v/v: 20%v/v) [27] as a solvent. The final dry extract (COE) was obtained *via* lyophilization and the percentage yield was calculated.

#### LC-MS analysis of cocoa beans extract

The following conditions were maintained in running the sample on LC-MS 2010A (Shimadzu Japan). The C18 column was used as a stationary phase and a 90:10 v/v ratio of methanol: water was used (flow rate of 200 μL min^−1^) as the mobile phase. The COE was dissolved in the mobile phase and injected (volume 5 μL) and absorbance was recorded at 254 nm.

### *In vitro* antioxidant assays

#### 2,2-diphenyl-1-picryl-hydrazyl-hydrate (DPPH) free radical scavenging assay

DPPH radical scavenging activity of the COE was carried out as explained by Brand- Williams et al [28]. Briefly, 0.1 mM DPPH solution was prepared, and from that 3.5 mL solution was added to 0.5 mL of different concentrations (20 to 100 μg/mL) of the COE in ethanol. The solution was shaken and kept at room temperature for 30min. The absorbance of the mixture was measured at 517 nm. Ascorbic acid was utilized as a standard. The effect of quenching on the percentage of DPPH was calculated using the following equation:

DPPH(%)=[A0−A1/A0]×100

where A0 is the absorbance of the control and A1 is the absorbance in the presence of the test.

#### Nitric oxide (NO) radical scavenging assay

NO generated from sodium nitroprusside (SNP) was measured as explained by Marcocci et al [29]. Griess reagent was prepared by adding 1% sulfanilamide in 2.5% phosphoric acid and 0.1% n- ethylenediamine dihydrochloride in 2.5% phosphoric acid. Briefly, 0.5 mL of 10 mM sodium nitroprusside in a phosphate-buffered salt solution was mixed with 1 mL of various concentrations of COE (50 to 800 μg/mL) and incubated for 180 min at 25°C. The COE was then mixed with a freshly prepared Griess reagent. The reaction mixture was transferred to a 96-well plate. Absorbance was quantified at 546 nm using a micro UV plate reader. Gallic acid was used as a positive control.

The percentage of nitrogen oxide scavenged (%) = [*A*0 − *A*1 / *A*0] × 100

Where A0 is the absorbance of the control and A1 is the absorbance in the presence of the test.

### *In vitro* cytotoxicity assay

#### Procurement of cell lines and their maintenance

Chinese Hamster Ovary (CHO) and Ehrlich Ascites Carcinoma (EAC) cell lines were procured from National Centre for Cell Sciences (NCCS), Pune and were cultured in Dulbecco’s Modified Eagle’s Medium (DMEM) supplemented with 10% fetal bovine serum (FBS), 20 μg/mL penicillin (100 U), and 100μg/mL streptomycin. The cells were sub-cultured and maintained until they reached 70% confluence in T25 flasks at 37°C with 5% CO_2_ in a humidified incubator.

#### Preparation of test samples

Initially, 10 mg/mL stock concentration of the COE and 5 mg/mL of hirsutrin was prepared in 5% DMSO in sterile water and filtered using a 0.22 μM syringe filter from which different concentrations (50–600 μg/mL for COE and 5 to 160 μg/mL for hirsutrin) were used for MTT assay. All the experiments were performed in triplicate.

#### MTT assay of COE and hirsutrin on EAC and CHO cell lines

The cytotoxic activity of COE and hirsutrin on EAC and CHO cell lines was determined using MTT assay [8]. The MTT assay is based on the reduction of yellow-colored water-soluble tetrazolium dye to formazan crystals. During the assay, cell lines were plated onto 96-well flat-bottom plates at a cell density of 20,000 cells/well, and the cells were allowed to grow for 24 h. The stock solutions of COE and hirsutrin were prepared in 5% DMSO. Then the cells were treated with COE and hirsutrin. The final volume in each well was made up to 250 μL with DMEM media supplemented with 3% FBS and incubated for 48 h (COE and hirsutrin) at 37°C in 5% CO_2_. Further, 20 μL of MTT reagent (5 mg/mL stock solution) was added to all the wells and incubated for 4h at 37°C in 5% CO_2_. After incubation, the wells were washed with PBS thrice to remove the MTT. The MTT reduction product (formazan crystals) was then dissolved in 100 μL of 99.5% DMSO by gentle shaking and the absorbance was noted at 570 nm using an ELISA plate reader. The cytotoxic activity was expressed as a percentage of cell viability in CHO and EAC cell lines compared with the control *i*.*e*. extract-treated *vs* untreated.

#### Hydrogen peroxide-induced oxidative stress in EAC and CHO cell line

Hydrogen peroxide was used for induction of oxidative stress as described by Balekar et al. [18] and Ponnusamy et al. [19]. The EAC and CHO cells were seeded at a density of 30,000 cells/well into a 96-well plate in DMEM supplemented with 10% FBS and incubated at 37°C, in a humidified 5% CO_2_ atmosphere overnight. A curve with H_2_O_2_ concentrations 0.0625, 0.125, 0.25, 0.5, and 1.0 mM was constructed to determine H_2_O_2_ concentration, decrease in cell viability by 50% after 24 h of exposure using MTT assay. Subsequently, EAC and CHO cells were seeded at a density of 30,000cells/well into a 96-well plate containing DMEM culture medium supplemented with 10% FBS and incubated overnight at 37°C, in a humidified 5% CO_2_ atmosphere. After 24 h, cells were pre-treated with hirsutrin and COE on both the cell lines. Later, after 12h of exposure with IC_50_ of H_2_O_2_ (0.1 mM for EAC and 0.13 mM for CHO), and percentage cytotoxicity was evaluated using MTT assay as detailed above.

### Statistical analysis

Network interaction was evaluated *via* edge count. Docking data are presented as energy in kcal/mol. Interaction stability and fluctuations through MD simulation were analyzed by RMSD and RMSF. All experimental data were presented in mean ± SD. The IC_50_ was calculated using a linear regression curve using GraphPad *ver* 5.

## Results

### Mining of bioactives and prediction of their targets

Fifty-four bioactives previously reported to be present in the cocoa were mined from the available phytochemical database and published literature (S1 Table). Among them, the majority of the bioactives were identified as flavonoids and phenols. Among the 54 bioactives, 34 were predicted to modulate 220 protein targets by BindingDB (S2 Table).

### Enrichment analysis and network construction

**Fig 1:**
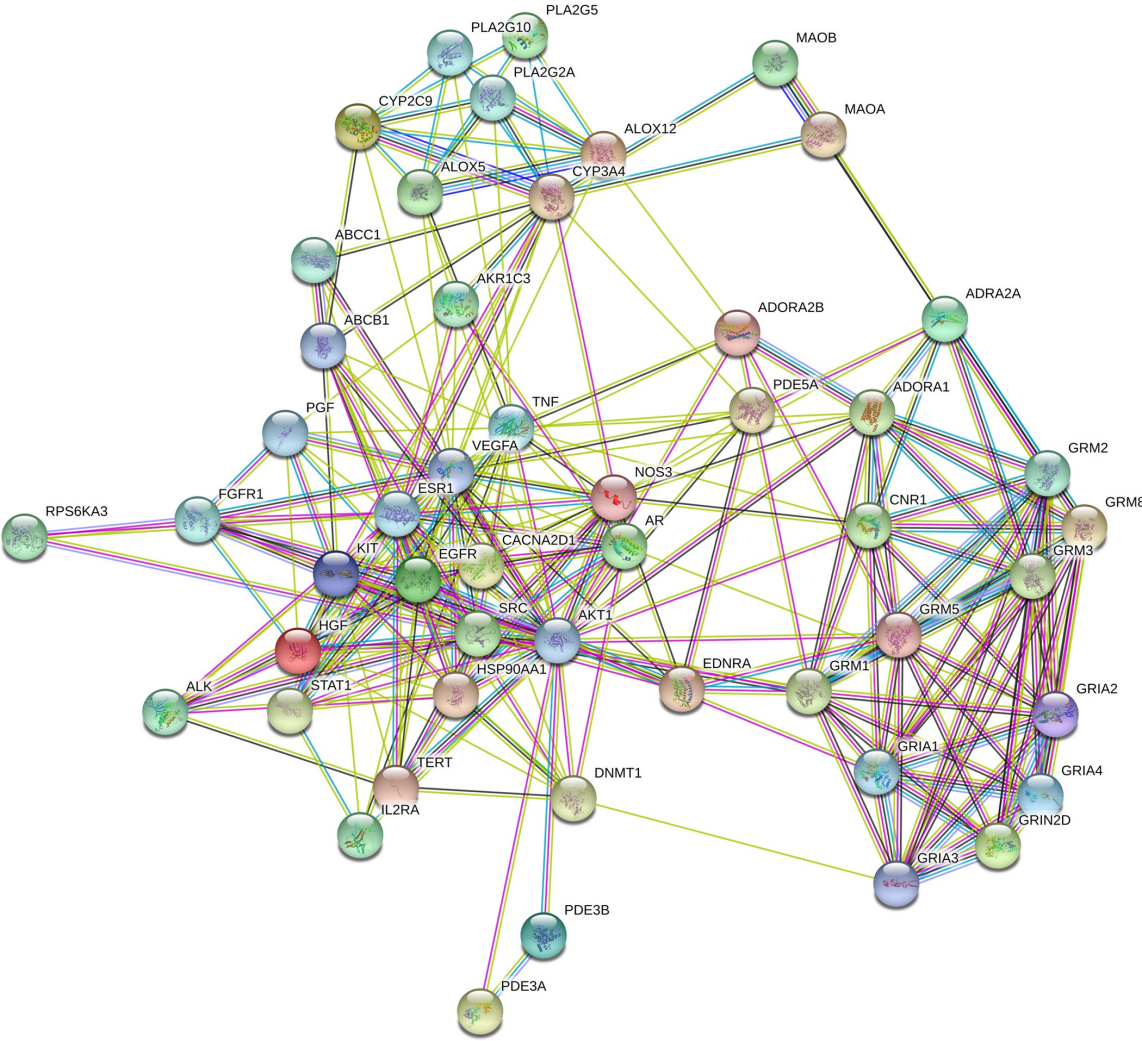
Protein-protein interaction of the regulated targets by the bioactives. 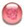
 colored nodes: Query proteins and first shell of interactors, 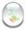 white nodes: Second shell of interactors, Node content; 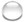 empty nodes: Proteins of unknown 3D structure, 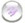 filled nodes: Some 3D structure is known or predicted.

Gene set enrichment analysis identified a total of 220 targets interacting with each other to regulate 104 pathways concerning the KEGG database (S3 Table). The probable protein-protein interaction of bioactives-regulated targets is presented in Fig 1. Among them, 21 pathways were identified to associate with oxidative stress and cancer via modulating 50 protein targets. The Arachidonic acid metabolism pathway (hsa00590) scored the lowest false discovery rate (FDR) of 1.27E-06 by triggering 7 genes (AKR1C3, ALOX12, ALOX5, CYP2C9, PLA2G10, PLA2G2A, and PLA2G5). Likewise, pathways in cancer (hsa05200) scored the second-lowest FDR of 1.34E-06 by modulating 15 genes (AKT1, ALK, AR, EDNRA, EGFR, ESR1, FGFR1, HGF, HSP90AA1, IL2RA, KIT, PGF, STAT1, TERT, and VEGFA). Following the cancer pathways, cAMP, Rap1, Ras, Phospholipase D, cGMP-PKG, MAPK, PI3K-Akt, and VEGF signaling pathways scored the lowest FDR and had potential involvement in oxidativeStress and cancer (Table 1).

**Table 1:** Enrichment analysis of pathways involved in oxidative stress and cancer.

Pathway ID	Pathway name	Gene count	False discovery rate	Regulated genes
hsa00590	Arachidonic acid metabolism	7	1.27E-06	AKR1C3, ALOX12, ALOX5, CYP2C9, PLA2G10, PLA2G2A, PLA2G5
hsa05200	Pathways in cancer	15	1.34E-06	AKT1, ALK, AR, EDNRA, EGFR, ESR1, FGFR1, HGF, HSP90AA1, IL2RA, KIT, PGF, STAT1, TERT, VEGFA
hsa04024	cAMP signaling pathway	10	1.67E-06	ADORA1, AKT1, EDNRA, GRIA1, GRIA2, GRIA3, GRIA4, GRIN2D, PDE3A, PDE3B
hsa04015	Rap1 signaling pathway	10	2.18E-06	ADORA2B, AKT1, CNR1, EGFR, FGFR1, HGF, KIT, PGF, SRC, VEGFA
hsa04014	Ras signaling pathway	10	5.60E-06	AKT1, EGFR, FGFR1, HGF, KIT, PGF, PLA2G10, PLA2G2A, PLA2G5, VEGFA
hsa04072	Phospholipase D signaling pathway	8	1.36E-05	AKT1, EGFR, GRM1, GRM2, GRM3, GRM5, GRM8, KIT
hsa04022	cGMP-PKG signaling pathway	8	2.38E-05	ADORA1, ADRA2A, AKT1, EDNRA, NOS3, PDE3A, PDE3B, PDE5A
hsa04010	MAPK signaling pathway	10	3.16E-05	AKT1, CACNA2D1, EGFR, FGFR1, HGF, KIT, PGF, RPS6KA3, TNF, VEGFA
hsa05205	Proteoglycans in cancer	8	7.57E-05	AKT1, EGFR, ESR1, FGFR1, HGF, SRC, TNF, VEGFA
hsa04151	PI3K-Akt signaling pathway	10	0.00011	AKT1, EGFR, FGFR1, HGF, HSP90AA1, IL2RA, KIT, NOS3, PGF, VEGFA
hsa01521	EGFR tyrosine kinase inhibitor resistance	5	0.00042	AKT1, EGFR, HGF, SRC, VEGFA
hsa05215	Prostate cancer	5	0.00095	AKT1, AR, EGFR, FGFR1, HSP90AA1
hsa04370	VEGF signaling pathway	4	0.0015	AKT1, NOS3, SRC, VEGFA
hsa00982	Drug metabolism—cytochrome P450	4	0.002	CYP2C9, CYP3A4, MAOA, MAOB
hsa05212	Pancreatic cancer	4	0.0026	AKT1, EGFR, STAT1, VEGFA
hsa05224	Breast cancer	5	0.0039	AKT1, EGFR, ESR1, FGFR1, KIT
hsa05226	Gastric cancer	5	0.0039	ABCB1, AKT1, EGFR, HGF, TERT
hsa05206	MicroRNAs in cancer	5	0.004	ABCB1, ABCC1, DNMT1, EGFR, VEGFA
hsa05219	Bladder cancer	3	0.0048	EGFR, SRC, VEGFA
hsa04923	Regulation of lipolysis in adipocytes	3	0.0084	ADORA1, AKT1, PDE3B
hsa04012	ErbBsignaling pathway	3	0.0228	AKT1, EGFR, SRC
hsa04630	Jak-STAT signaling pathway	4	0.0228	AKT1, EGFR, IL2RA, STAT1

**Fig 2:**
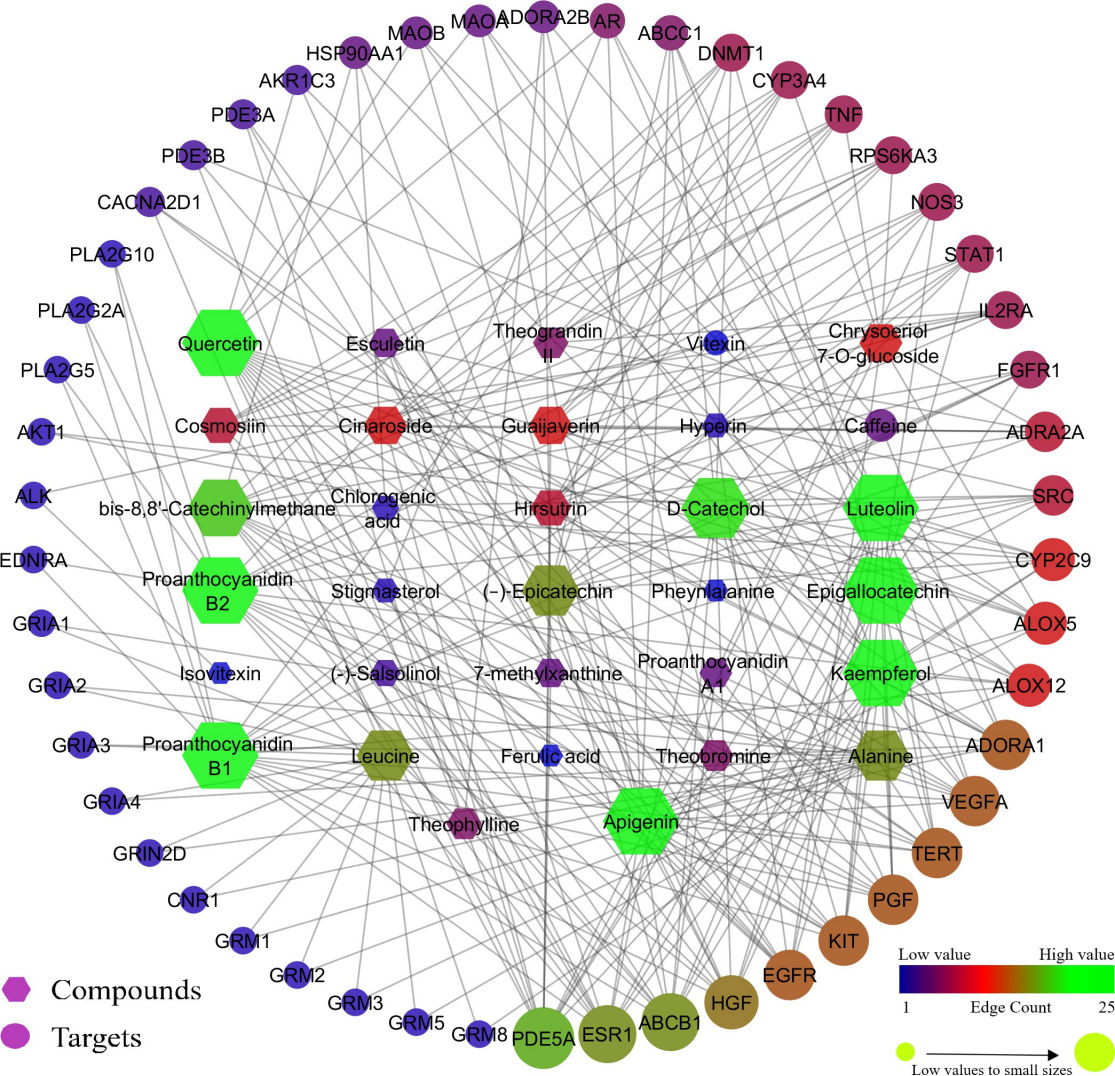
Interaction of phytocompounds with respective predicted targets. Hexagon represents the compounds and circle represents the targets.

**Fig 3:**
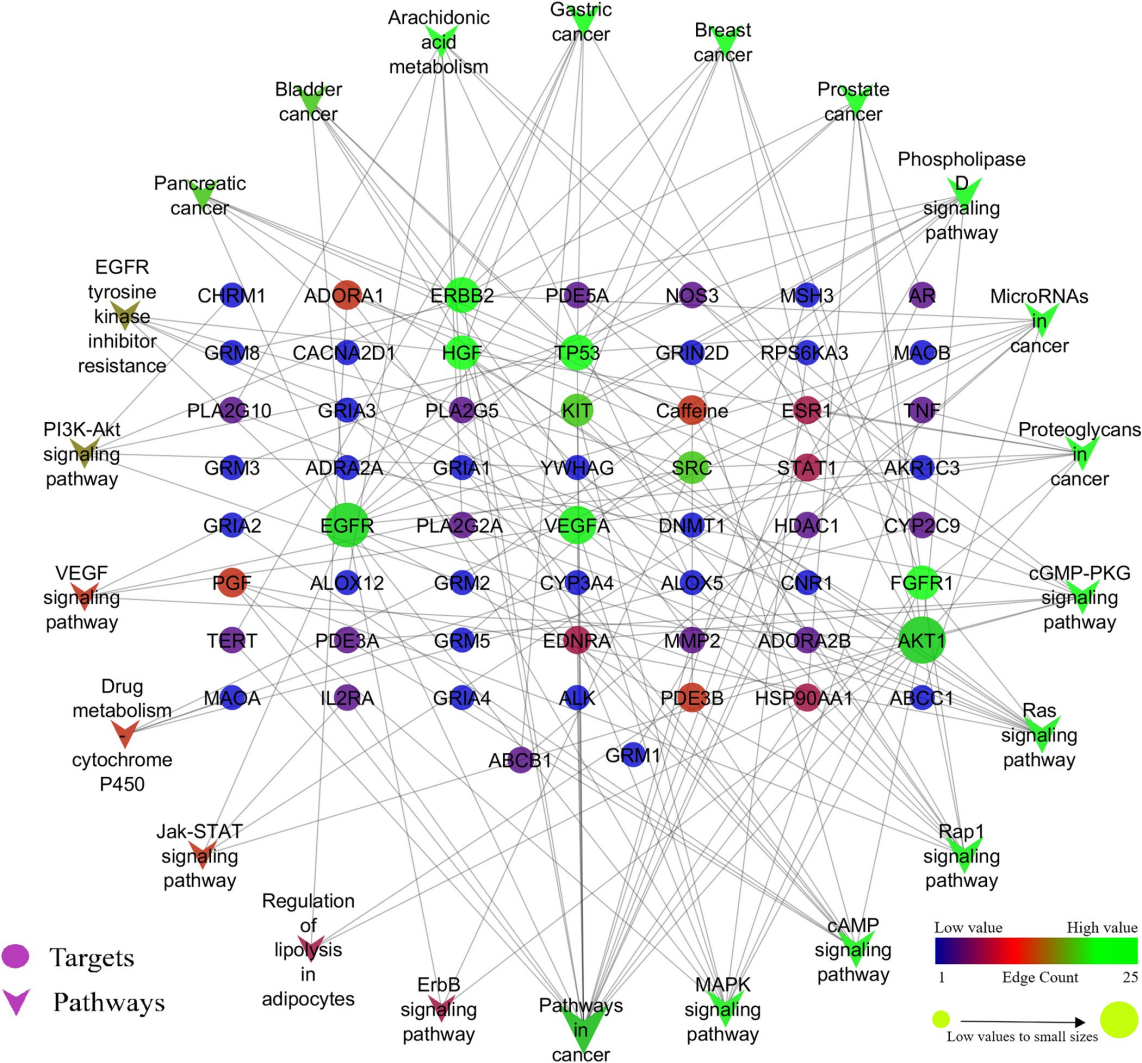
Pathways regulated and their representative proteins concerning the KEGG database. Arrow represents the modulated pathways and circle represents the targets.

**Fig 4:**
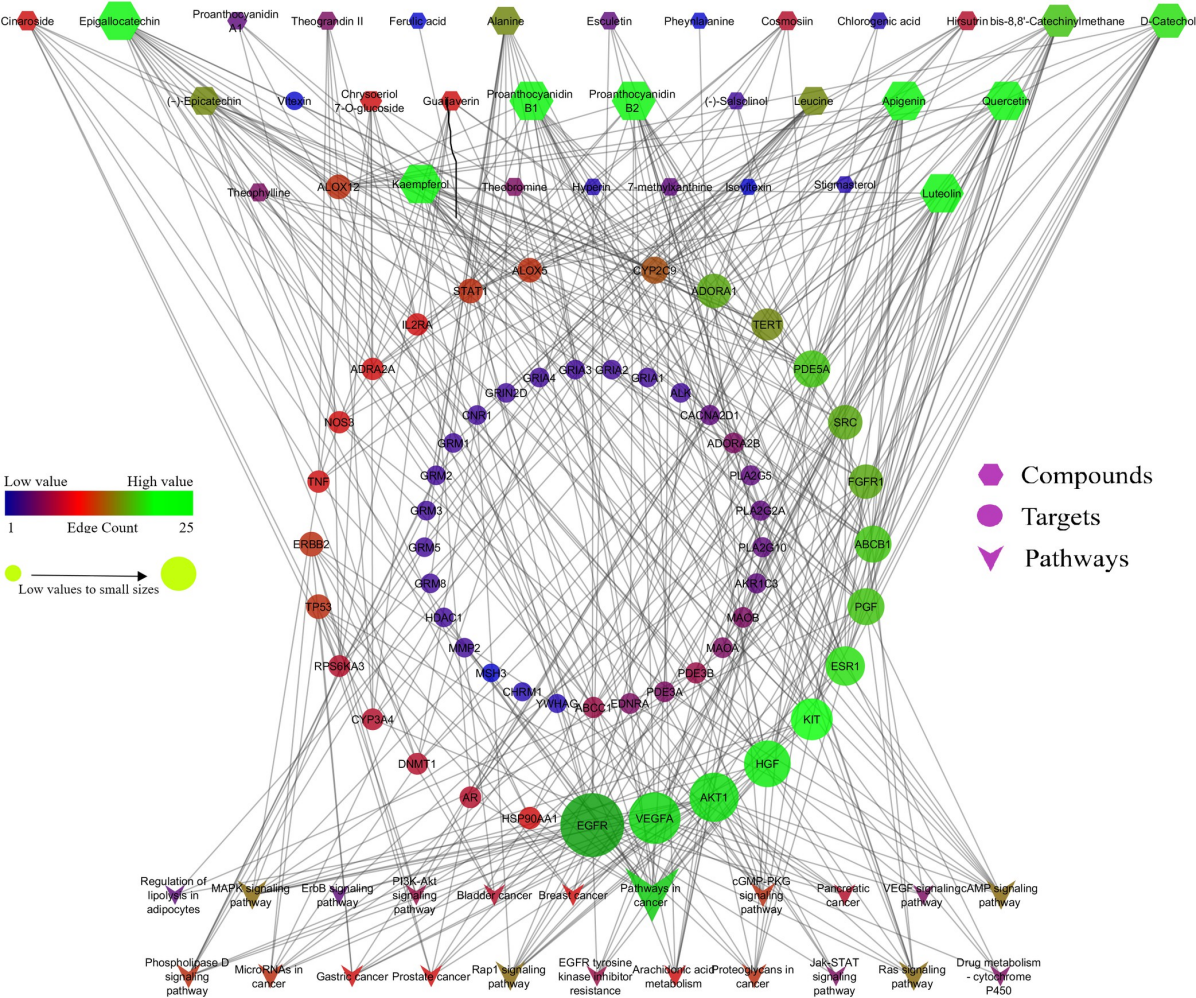
Interaction of the bioactives with their targets and modulated pathways. Hexagon represents the compounds, circle represents the targets, and arrow represents the pathways.

The network between compound-protein (Fig 2), protein-pathway (Fig 3), and compounds-proteins-pathways (Fig 4) were constructed by treating edge count topological parameters using Cytoscape *ver* 3.6.1. Network analysis identified, among all the queried protein targets involved in cancer and oxidative stress, EGFR was identified as an enriched hub protein within the network that scored the highest edge count (Fig 4). EGFR was found to involve in 15 pathways out of 21 and targeted by 11 bioactives of cocoa. Based on network analysis, EGFR was selected to infer the intermolecular interactions with bioactives of cocoa by molecular docking and dynamics analysis.

### EGFR structure refinement, lowest PE conformation from MD, and its active site residues

**Fig 5:**
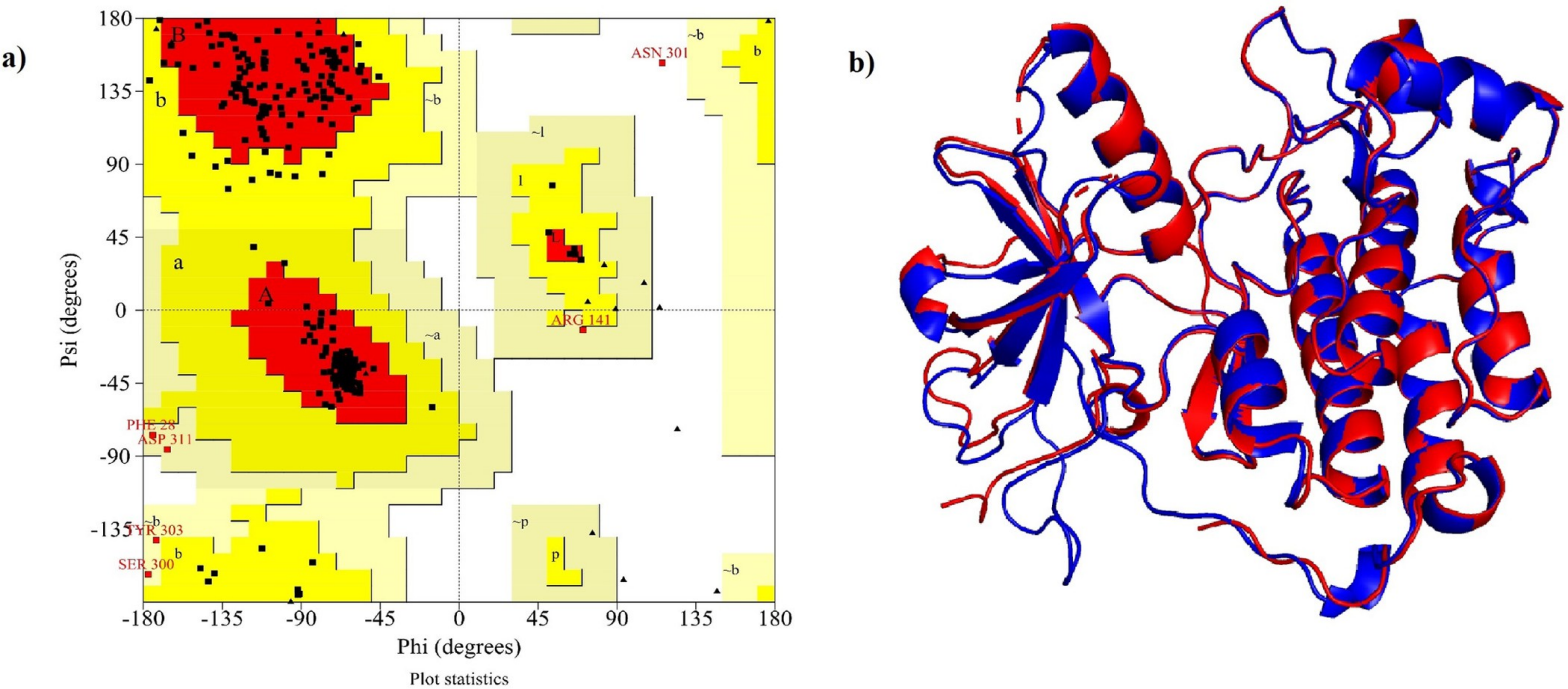
**(a) Ramachandran plot of EGFR protein.** Red region represents the most favoured region,Dark yellow represents the favoured region, fade yellow represents the allowed region and the white region represents the non-allowed region. **(b) 3D structure of EGFR generated using homology modeling.** Blue represents modeled protein, red represents template (PDB: 6LUB).

**Fig 6:**
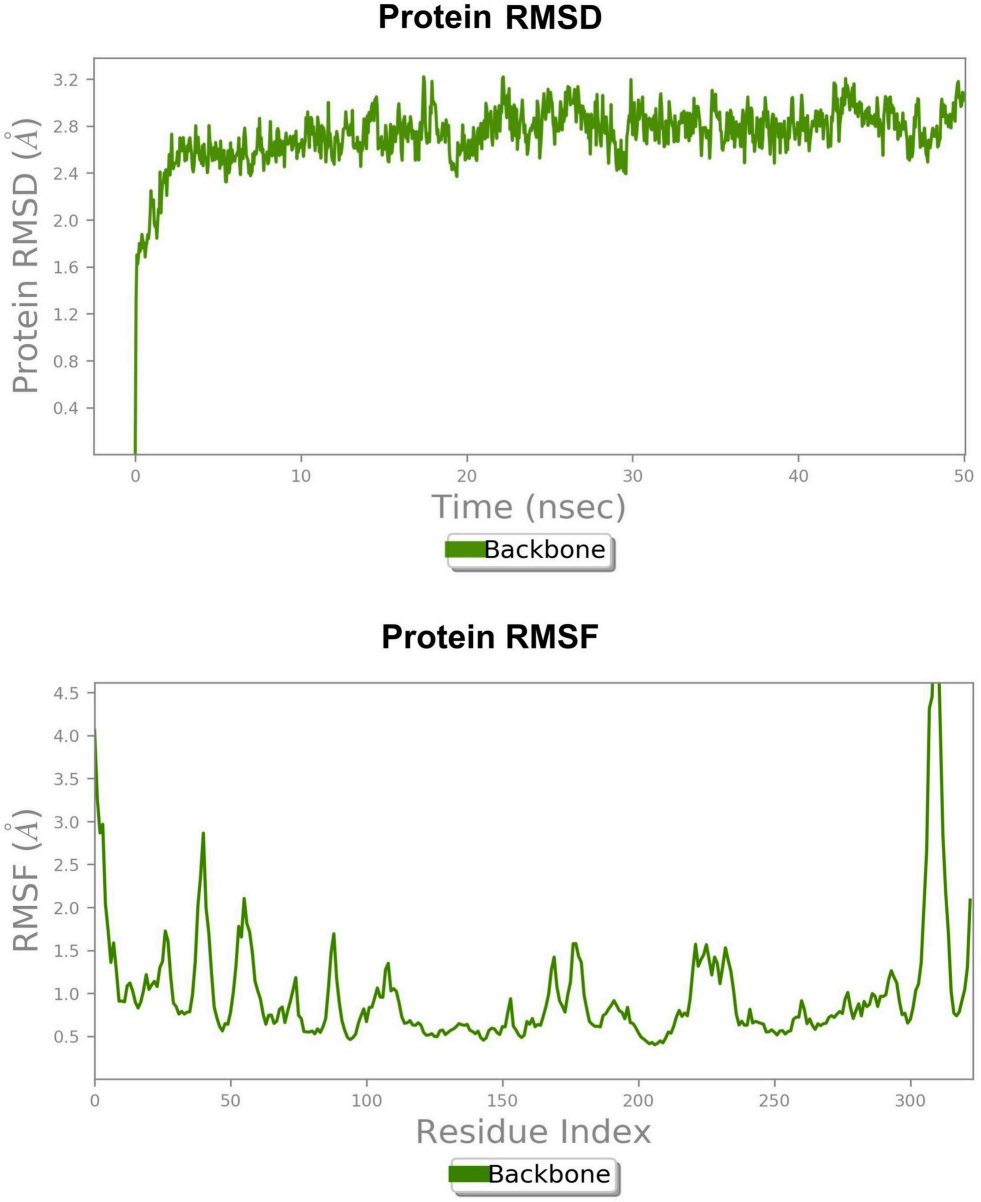
Structural stability of the generated homology model of EGFR protein observed during 50ns MD simulations. (a) root mean square fluctuation revealed EGFR reached equiliberation state after ~22 ns and showed stable dynamics thereafter. (b) binding pocket residues Leu144, Ala48, Ser102, Val31, Met98, Cya33, Pro99, Tyr106 etc. showed less residual fluctuations however other flexible loop regions including N and C-terminal residues show maximum residual fluctuations.

A total of 100 models were generated of which we select model 63 as it shows the least DOPE score (-37918.11). The stereochemical properties of the modeled EGFR were analyzed by generating a Ramachandran plot (Fig 5A). The RMSD of 0.359 Å with template revealed the reliability of the selected model (Fig 5B). The parameters describing structural stability such as RMSD and RMSF revealed stable dynamics during the 50 ns simulation (Figs 6A and 4B respectively). Further, the structure with the least potential energy was extracted at 33.7 ns from the MD simulation trajectory and used for docking study.

### Molecular docking

**Fig 7:**
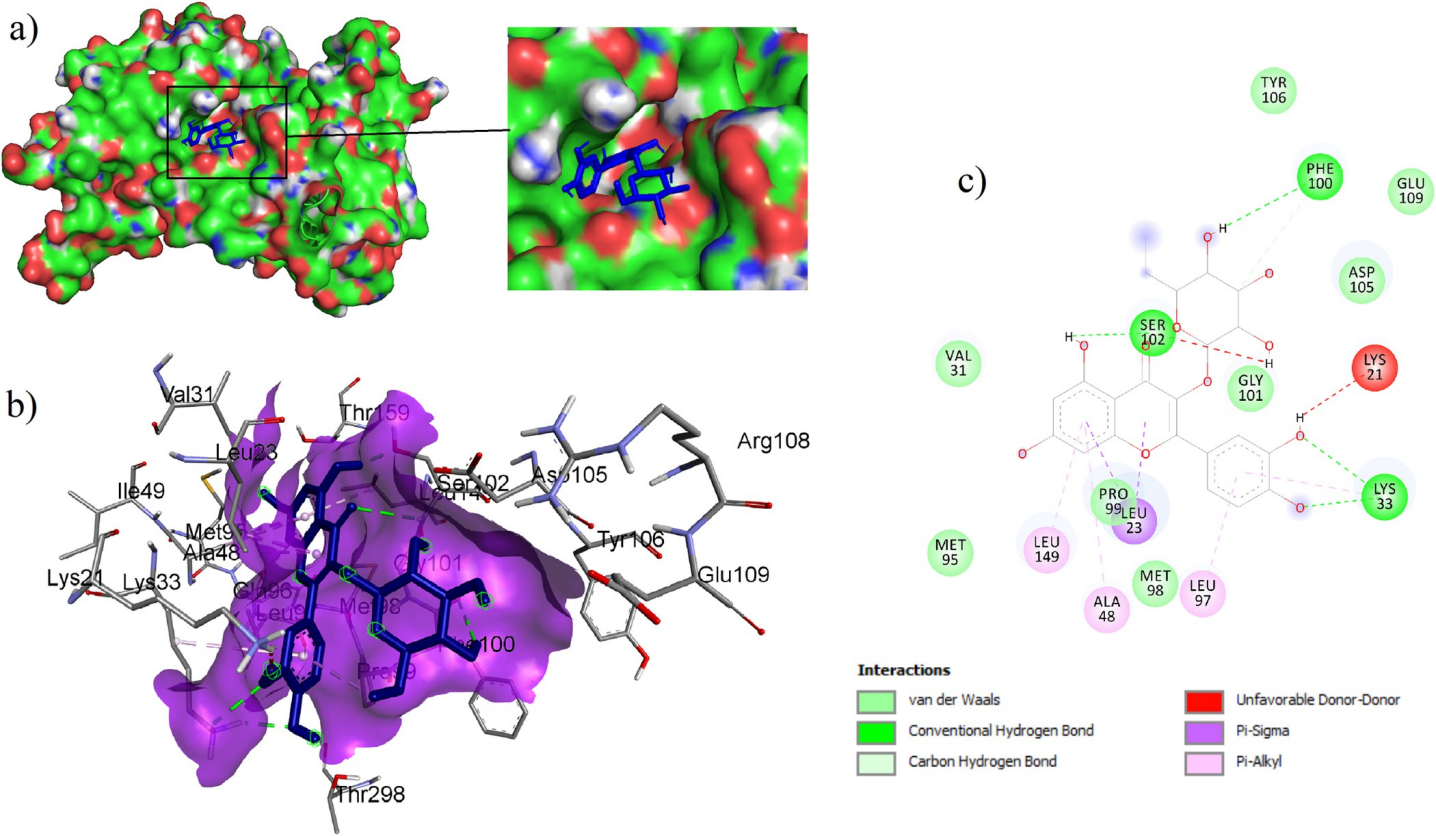
(a) Surface view of EGFR showing ligand binding sites where hirsutrin is deeply buried (refer inset) in to the EGFR binding cavity forming compact globular shape and (b) Intermolecular interactions observed in complex EGFR-Hirstutrin.

The active site residues of EGFR namely Leu23, Lys33, Ala48, Lys50, Cys80, Met95, Gln96, Leu97, Met98, Pro99, Arg146, Asn147, Leu149, Thr159, and Asp160 were taken from the crystal structure “6LUB.pdb”. The docking analysis revealed all 11 compounds were efficiently bound to the EGFR binding pocket. Amongst them, hirsutrin (BE is -7.2 kcal/mol), also known as isoquercitrin showed a maximum number of stable interactions (11), of which 8 interactions were precise with the defined binding pocket residues (Fig 7). We observed H- bonding interactions with Ser102, Phe100, Lys33, and other non-bonded interactions with Lys33, Leu97, Ala48, Leu149, Leu23, and Phe100. However, apigetrin showed the least binding energy with EGFR (- 9.0kcal/mol) by forming a maximum of 8 interactions, of which only 4 interactions are with the active site residues Arg146, Asn147 (2), Lys50. Erltonib shows BE of -7.0 forming two H-bonds with active site residues Asn147 and Lys50. It also formed six non-hydrogen bonds with residues Ala144, Trp185, Gly162, Glu211, and Phe28 (2). Therefore we select Hirsutrin-EGFR for further MD simulation study. Table 2 lists the binding energy of all the docked bioactives and their intermolecular interactions.

**Table 2:** Binding affinity of compounds with EGFR.

Compound name	Compound CID	BE (kcal/mol)	Conventional HBI	Non-conventional interactions	No. of interactions with active site residues
Apigetrin	5280704	-9.0	Asn147 (2), Arg146	Glu67, Leu52 (2), Ile64, Lys50	4
Cinaroside	5280637	-8.6	Glu211, Arg146 (2), Lys50	Trp185 (2), Ala144 (2), Arg146 (2)	5
Chrysoeriol7-O-glucoside	11294177	-8.3	Arg146, Glu67 (2)	Trp185, Ala144 (2), Arg146 (3), Phe28	4
Kaempferol	5280863	-8.0	Phe28, Arg146	Leu52 (2), Ile64, Lys50, Glu67 (2), Arg163, Gly162, Ile64	2
Quercetin	5280343	-7.8	Nil	Val31, Leu149, Leu23	2
Apigenin	5280443	-7.7	Arg146	Glu67 (2), Leu52 (2), Lys50, Ile64	2
Luteolin	5280445	-7.7	Asp160, Met98	Leu149, Ala48, Val31, Leu23 (2)	6
Hirsutrin	74982342	-7.2	Ser102, Phe100, Lys33 (2)	Lys33, Leu97, Ala48, Leu149, Leu23 (2), Phe100	8
Hyperosid	90657624	-7.2	Ser102, Asp105, Lys33	Leu149, Leu23 (2), Ala48, Phe100	5
Ferulic acid	445858	-6.4	Arg146.O- (2), Met98…OH, Met98… = O	Leu149, Leu23, Val31, Asp160	7
Esculetin	5281416	-6.2	Leu23, Met98	Gly101, Ala48, Leu149, Leu23 (2)	6

BE, Binding energy; HBI, Hydrogen bond interactions.

### Hirsutrin-EGFR complex MD simulation

**Fig 8:**
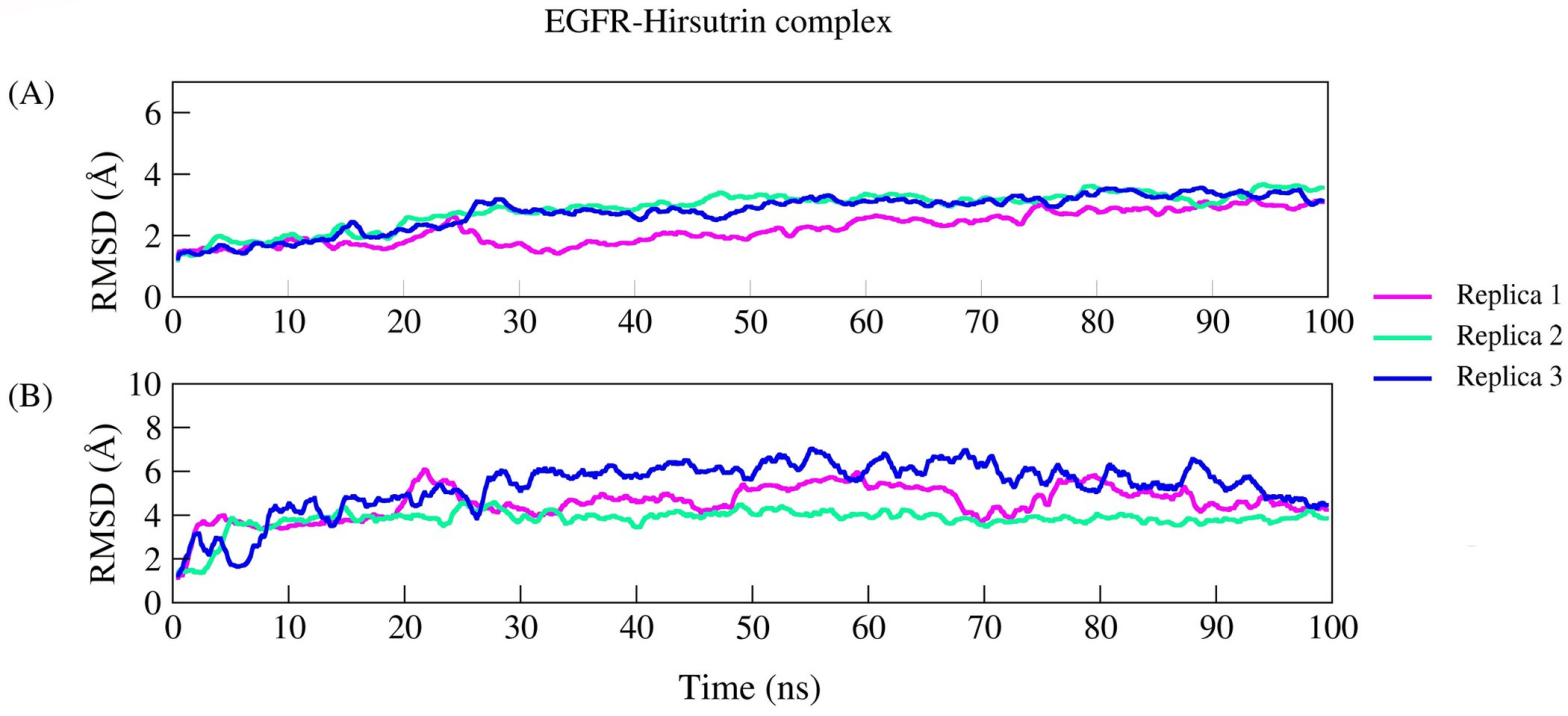
(a) Root mean square deviation of EGFR backbone, and (b) hirsutrin in complex with EGFR.

**Fig 9:**
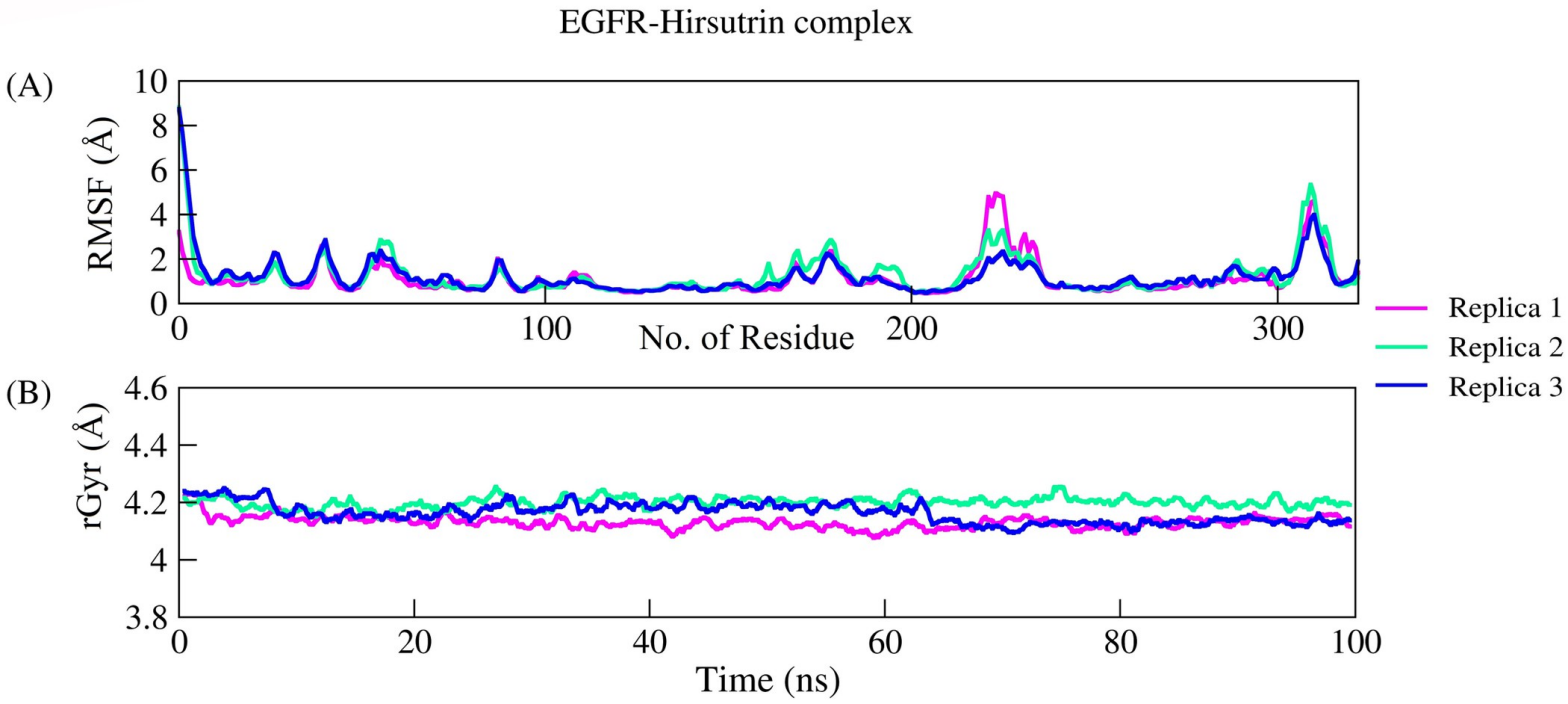
(a) The qualitative parameters explaining structural stabilities like residual fluctuations and (b) radius of gyration in all the replicas.

The complex Hirsutrin-EGFR exhibited stable dynamics as revealed by parameters RMSD, RMSF, rGyr, and intermolecular interactions in all three replicas. Backbone EGFR showed a mean RMSD of 1.18Å and 3.22Å between the initial and final frame. The average backbone RMSD for all three replicas was 2.62Å. The complex RMSD was 4.56Å (initial and final frame RMSD was 1.094Å and 4.281Å, respectively). Further, the residue-wise fluctuation was analyzed through RMSF for the protein backbone. The average RMSF of protein residues (322 residues) was 1.20Å. The c- terminal loop residues (300–320) showed maximum fluctuation for ~5.5Å due to the flexibility. Active site residue involved in the interactions viz., Gln96, Met98, and Asp105 showed the least RMSF fluctuation (~1.5Å) in all three replicas throughout the simulation. The ligand means rGyr for three replicates was 4.16Å (initial and final frame rGyr was 4.26Å and 4.144Å, respectively). The overall residual fluctuations of EGFR were very less except terminal residues and loop regions. The complex Hirsutrin-EGFR formed a compact globular shape as revealed by a steady decrease in the rGyr values. Fig 8 represents the RMSD of the EGFR backbone (8A) and complex (8B). Fig 9 represents the RMSF of protein (9A) and ligand rGyr fluctuation (9B). Residues Gln96, Met98, and Asp105 show very stable and conserved interactions throughout the simulation period in all three replicates. In replica 1, Met98 showed an interaction fraction of about 53% with hirsutrin whereas, in replicas 2 and 3, it showed interaction fractions of 91% and 89% respectively. Similarly, another important binding pocket residue Gln96 showed about 52%, 65%, and 88% interaction fraction in replicas 1, 2, and 3, respectively with hirsutrin.

### Drug-likeness and side effects

All the compounds scored a positive DLS except ferulic acid and esculetin (Table 3). Interestingly, hirsutrin scored the highest DLS of 0.84 which showed stable and maximum interactions in docking and MD simulations. While apigenin and luteolin scored the least DLS of 0.39 and 0.38 respectively. The possible side effect is predicted using the ADVERpred server. Four compounds namely, apigenin, quercetin,luteolin, kaempferol scored Pa>0.5 for hepatotoxicity, 6 compounds ferulic acid, cinaroside, hirsutrin, esculetin, chrysoeriol7-o- glucoside, and apigetrin scored Pa<0.5 for hepatotoxicity, nephrotoxicity, and myocardial infarction. A compound hyperosid did not show any probable side effects. Similarly, a control molecule, erlotinib was also predicted to cause hepatotoxicity (Pa = 0.90), myocardial infarction (Pa = 0.665), nephrotoxicity (Pa = 0.594), and cardiac failure (Pa = 0.362).

**Table 3:** Druglikeness and side effects profile of bioactives targeting EGFR.

Cocoa bioactives	MF	MW (g/mol)	NHBA	NHBD	LogP	DLS	Pa	Pi	Predicted side effect(s)
Apigenin	C_22_H_18_O_10_	442.4	5	3	3.06	0.39	0.525	0.182	Hepatotoxicity
Apigetrin	C_21_H_20_O_10_	432.4	10	6	0.31	0.59	0.38	0.096	Nephrotoxicity
							0.363	0.288	Hepatotoxicity
Chrysoeriol7-O-glucoside	C_22_H_22_O_11_	462.4	11	6	0.28	0.56	0.361	0.108	Nephrotoxicity
Cinaroside	C_21_H_20_O_11_	448.4	11	7	-0.07	0.6	0.395	0.262	Hepatotoxicity
							0.373	0.1	Nephrotoxicity
Esculetin	C_9_H_6_O_4_	178.14	4	2	1.08	-1.22	0.463	0.216	Hepatotoxicity
Ferulic acid	C_10_H_10_O_4_	164.16	3	2	2.07	-0.61	0.44	0.057	Myocardial infarction
Hirsutrin	C_21_H_20_O_12_	464.4	12	8	-0.64	0.84	0.387	0.268	Hepatotoxicity
Kaempferol	C_15_H_10_O_6_	286.24	6	4	2.49	0.5	0.525	0.182	Hepatotoxicity
Luteolin	C_15_H_10_O_5_	270.24	6	4	2.68	0.38	0.559	0.165	Hepatotoxicity
Quercetin	C_15_H_10_O_7_	302.23	7	5	2.11	0.52	0.559	0.165	Hepatotoxicity
Hyperosid	C_21_H_19_O_12_	463.09	12	7	-0.32	0.64	Not predicted		

MF, Molecular Formula; MW, Molecular Weight, HBA, Hydrogen Bond Acceptor; HBD, Hydrogen Bond Donor; LogP, Partition Co-efficient; DLS, Druglikeness score; Pa, Probable activity; Pi, Probable inactivity.

### Extraction and LC-MS profile of COE

**Fig 10:**
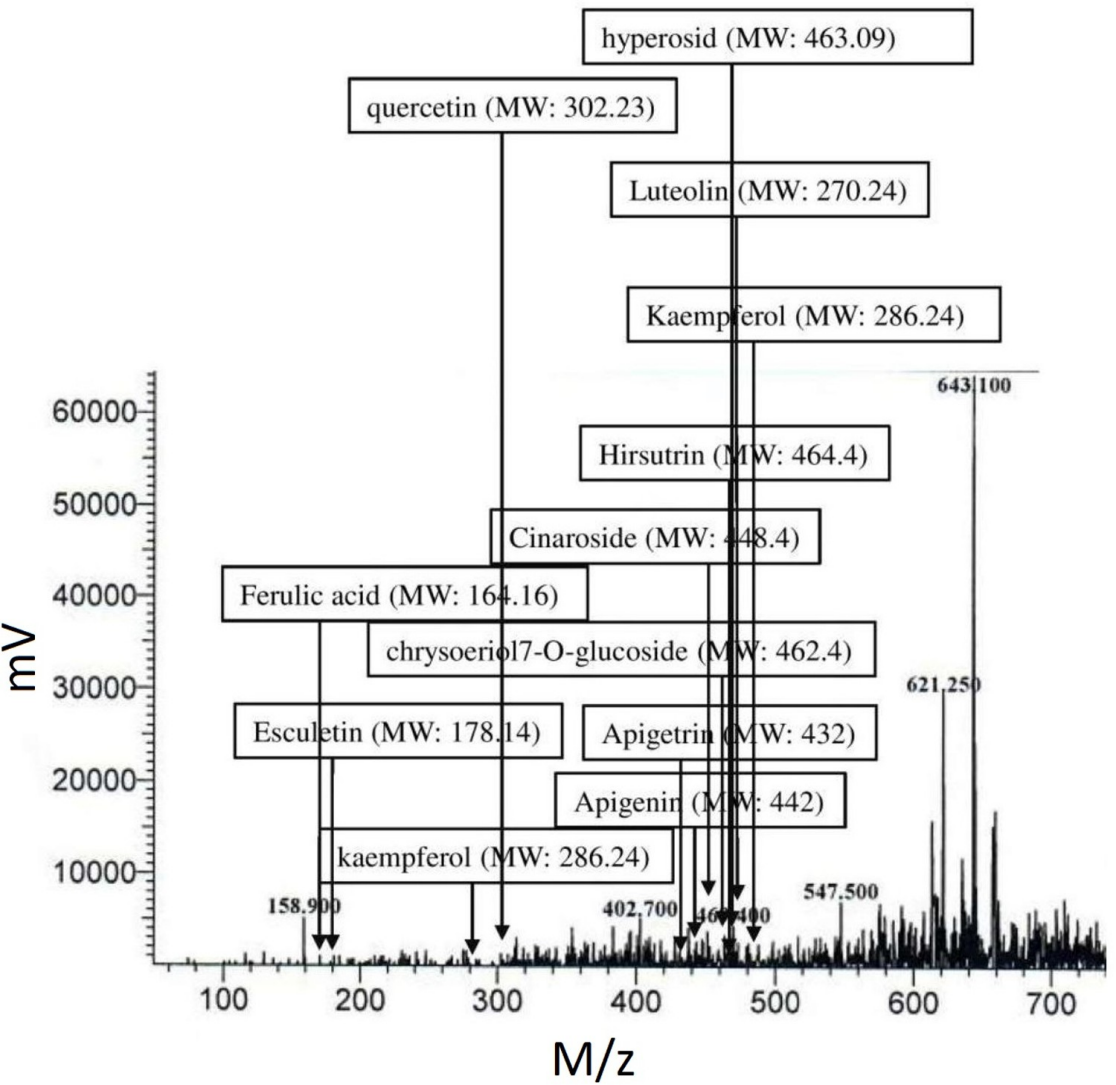
LC-MS analysis of COE.

The yield of the hydroalcoholic extract was 5.8% w/w of the dried cocoa bean. LC-MS profile of cocoa bean extract also traced the presence of active principles which were incorporated in the network pharmacology. Some of the lead hit bioactives with positive drug-likeness score and identified in the LC-MS analysis includes ***apigenin*** (MF: C_22_H_18_O_10_, MW: 442.4), ***apigetrin*** (MF: C_21_H_20_O_10_, MW: 432.4), ***chrysoeriol7-O-glucoside*** (MF: C_22_H_22_O_11_, MW: 462.4), ***cinaroside*** (MF: C_21_H_20_O_11_, MW: 448.4), ***esculetin*** (MF: C_9_H_6_O_4_, MW: 178.14), ***ferulic acid*** (MF: C_10_H_10_O_4_, MW: 164.16), ***hirsutrin*** (MF: C_21_H_20_O_12_, MW: 464.4), ***kaempferol*** (MF: C_15_H_10_O_6_, MW: 286.24), ***luteolin*** (MF: C_15_H_10_O_5_, MW: 270.24), ***quercetin*** (MF: C_15_H_10_O_7_, MW: 302.23), and ***hyperosid*** (MF: C_21_H_19_O_12_, MW: 463.09); Fig 10.

### In-vitro anti-oxidant assays

#### DPPH and Nitric Oxide free radical scavenging assay

**Fig 11:**
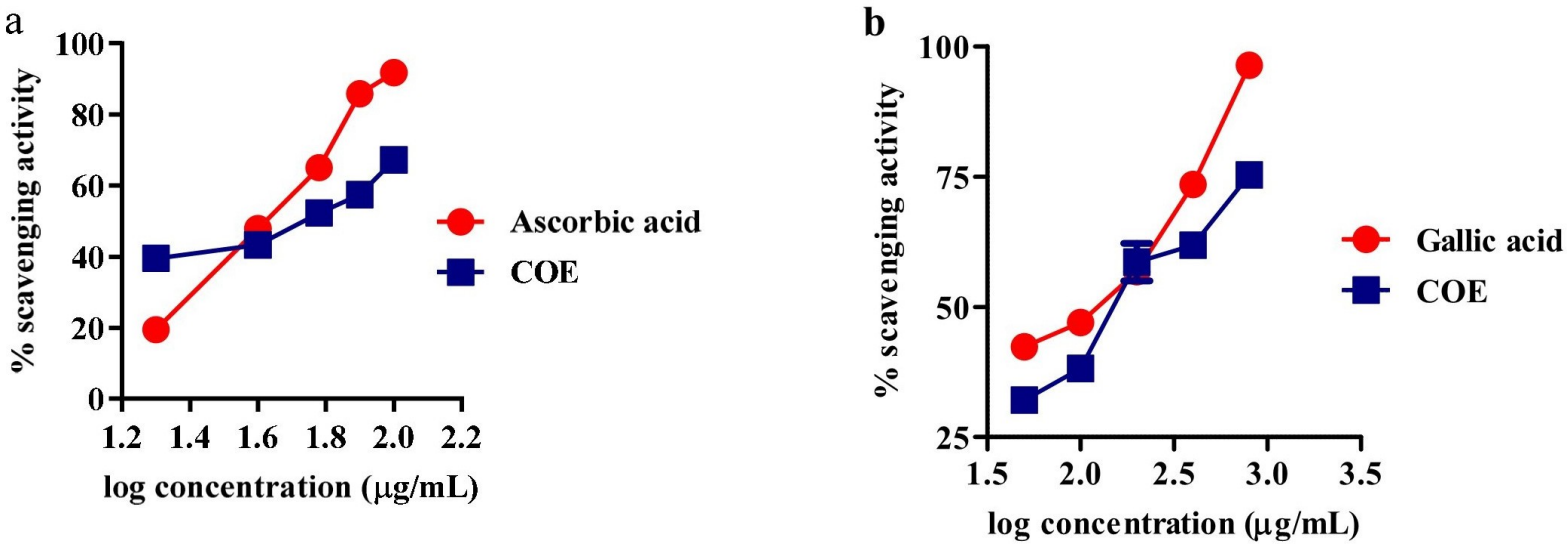
(a) DPPH radical and (b) nitric oxide scavenging activity of COE and ascorbic acid.

For DPPH assay, the IC_50_ of the extract was found to be (54.35±0.09) μg/mL, whereas, IC_50_ of ascorbic acid was (46.84±1.164) μg/mL. Further, for nitric oxide assay, the IC_50_ of the COE was (249.82±36.42) μg/mL, whereas, the IC_50_ of gallic acid was (125.96±6.41) μg/mL; Fig 11.

### In vitro cytotoxicity assay: MTT assay

**Fig 12:**
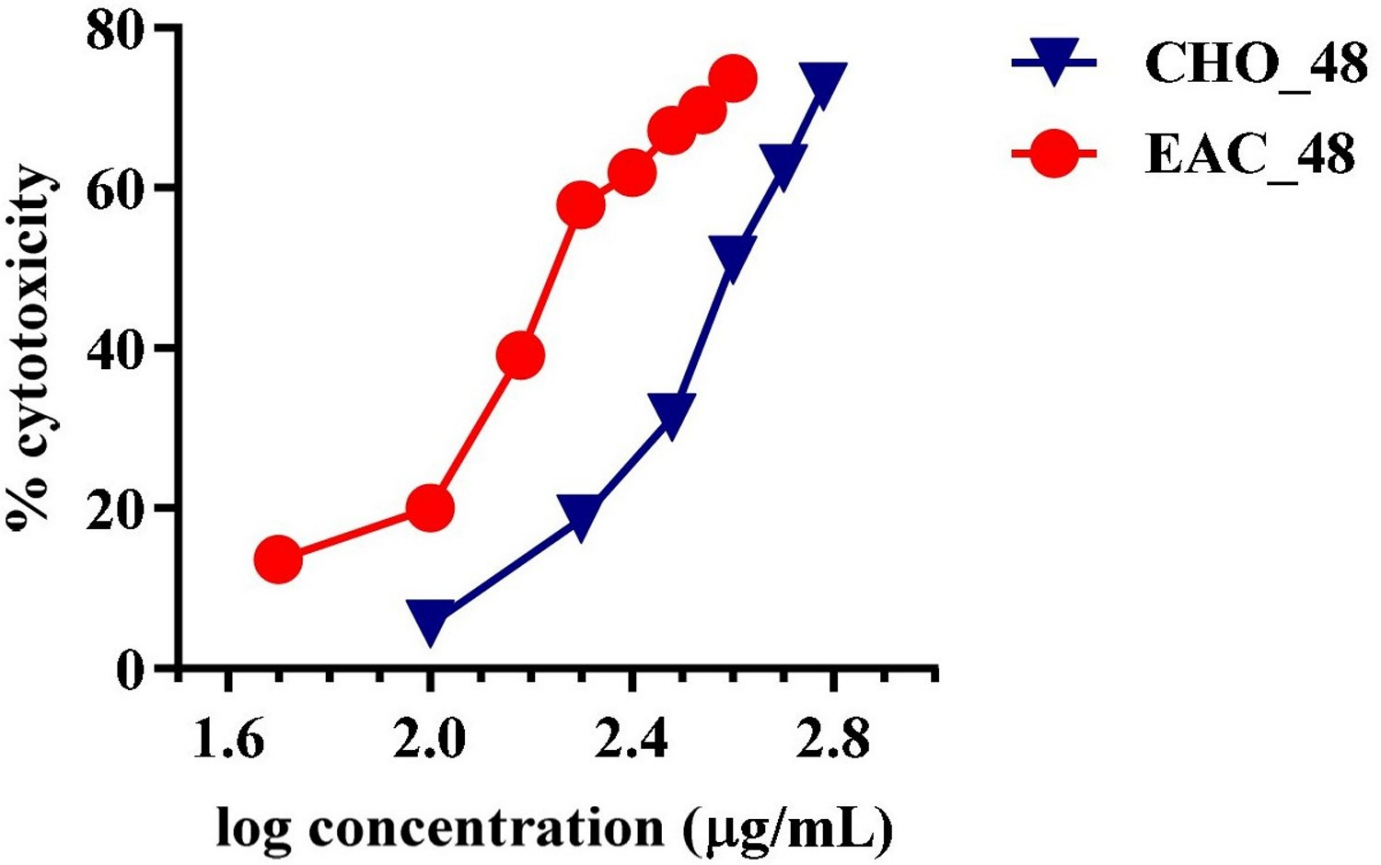
Cytotoxicity of COE in CHO and EAC cancer cell line after 48 h treatment.

**Fig 13:**
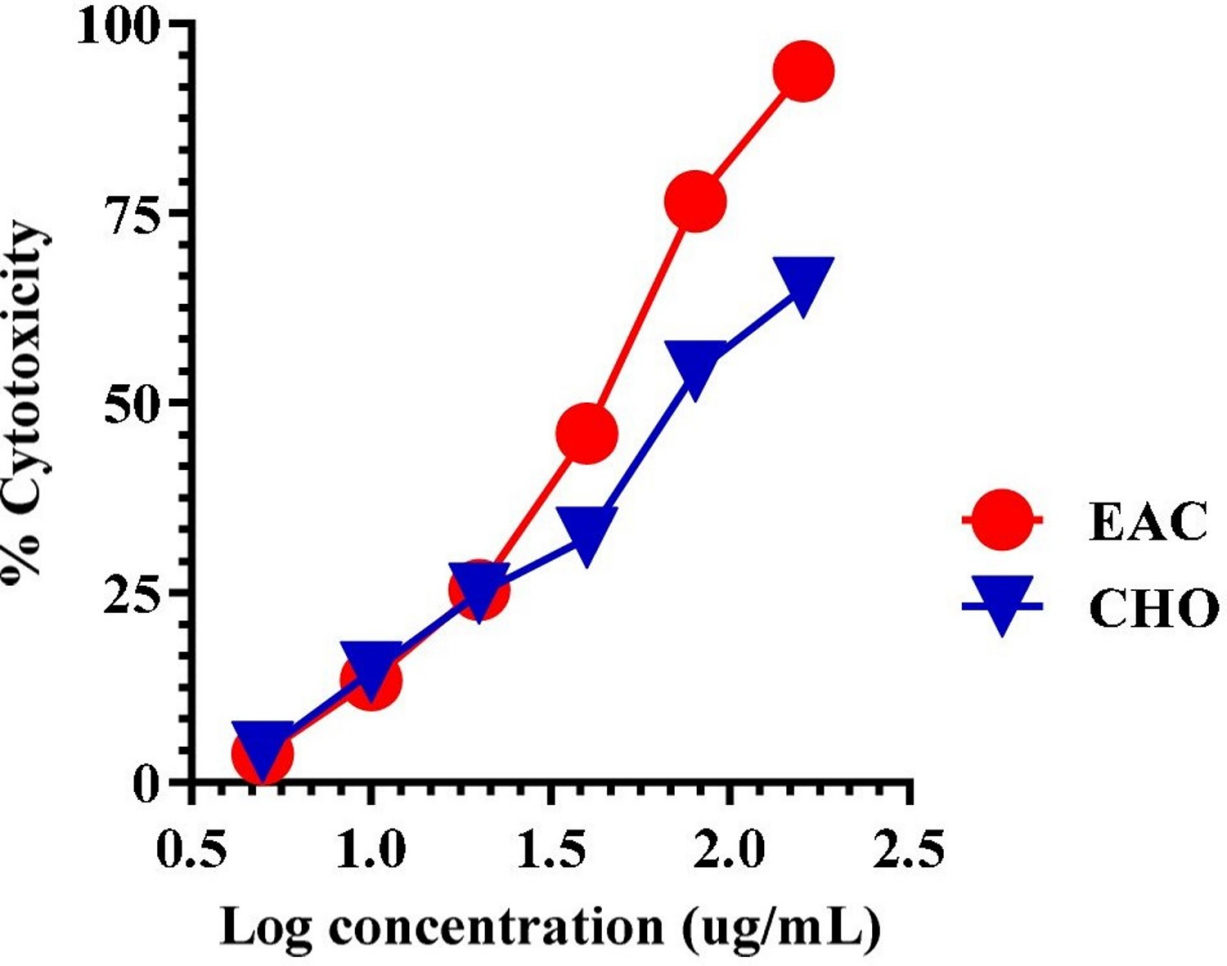
Cytotoxicity of hirsutrinin CHO normal cell line and EAC cancer cell line after 48 h treatment.

Cytotoxic activity of COE (Fig 12) and hirsutrin (Fig 13) was performed in CHO and EAC. The mean IC_50_ of the cocoa extract against CHO and EAC cell at 48h was 420.15±5.4μg/mL and 222.8±0.68μg/mL, respectively. Further, the mean IC_50_ of the hirsutrin against CHO and EAC cells were found to be 100.27±0.87μg/mL and 64.79±1.74μg/mL, respectively.

### Hydrogen peroxide-induced oxidative stress in EAC and CHO cell line

**Fig 14:**
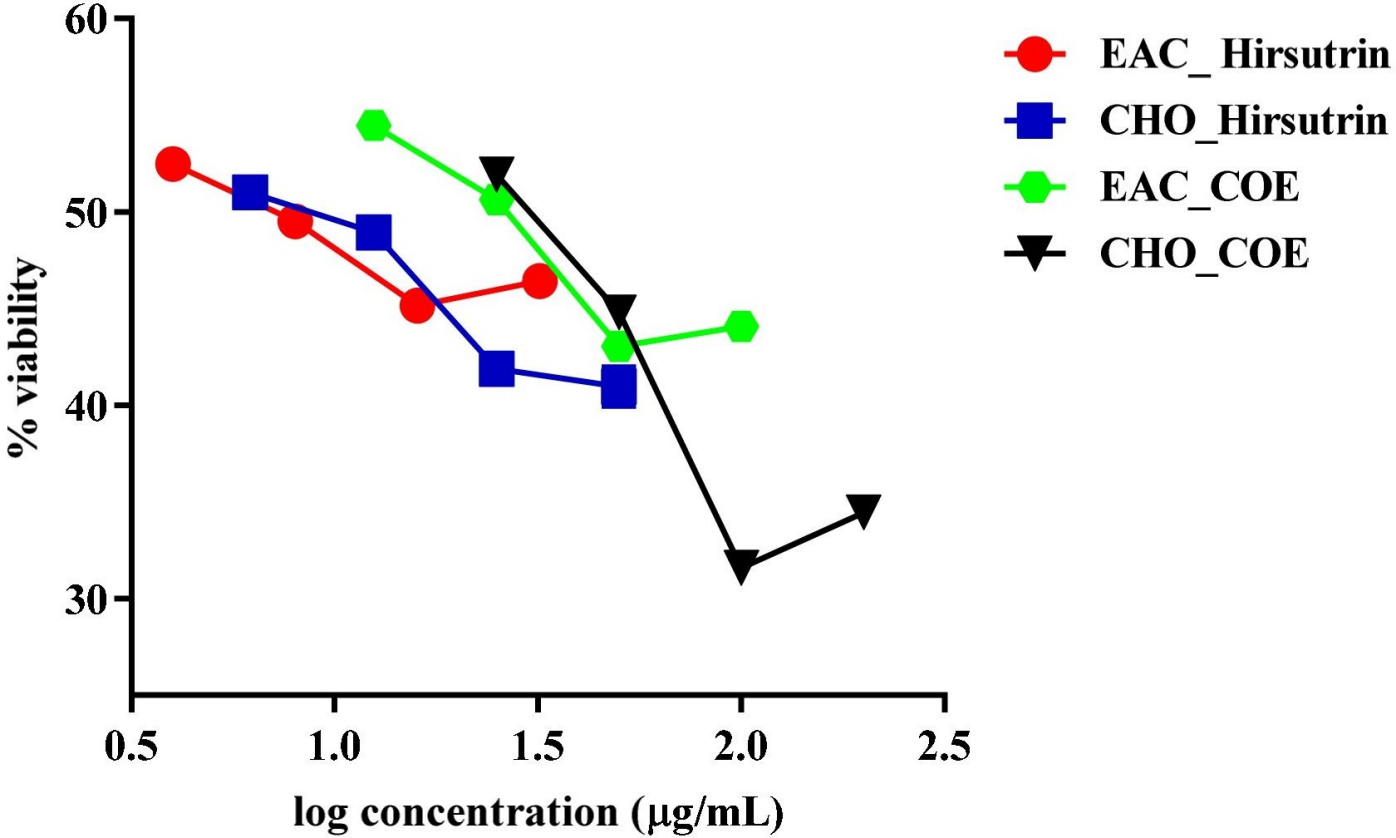
Cytotoxicity on EAC and CHO cells treated with hirsutrin and COE after H_2_O_2_ exposure.

Cells were treated with IC_50_ value of H_2_O_2_ respective to the EAC and CHO cell lines which resulted in decrease of cell viability by 50% after 24 h exposure. Similarly, when cells were pre-treated with COE and hirsutrin for 24 h, followed by IC_50_ value of H_2_O_2_ exposure for 12 h showed a protective effect against H_2_O_2_-triggered oxidative stress; maintained the cell viability (Fig 14).

## Discussion

The present study traced 54 documented bioactives of cocoa to propose a probable mechanism against oxidative stress and cancer pathogenesis. The bioactives were predicted to target 220 proteins and were found to involve in 104 pathways, in which 50 targets and 21 pathways were found to associate with oxidative stress and cancer. The network of interactions between bioactives, protein molecules, and their pathways was constructed and analyzed based on ‘edge count’ to identify the node with maximum interactions that indicate key molecules [30]. Among 50 targets predicted, EGFR was identified as a major druggable target for cocoa bioactives, in which EGFR modulated in 15 pathways within the network. Out of 34 bioactives, 11 were identified to target EGFR. EGFR is a potent oncogene frequently overexpressed in a variety of cancers and is already a well-known therapeutic target in cancer therapy [31]. EGFR inhibitors have beneficial effects against cell proliferation and progression in a wide variety of cancer types [32]. Catechins from green tea extract have been reported to prevent colon cancer and hepatocellular cell carcinoma by blocking the activation of the RTKs, primarily EGFR, IGF‐ 1R, VEGFR2, and related pathways [33]. Cocoa polyphenols exhibit potent antioxidant, anti-inflammatory, and chemo-protective effects by reducing TNF-α-induced up-regulation of VEGF by directly inhibiting PI3K and MEK1 activities [34]. Further, an *in vitro* study demonstrated a combination of glucose-(-)-epigallocatechin-3- gallate derivatives, and chemotherapeutics as a treatment for non-small cell lung cancer [35]. Epicatechin, an antioxidant flavonoid regulates nitric oxide production and exhibits anti-inflammatory effects in addition to cardiovascular protective effects on vascular endothelium [14]. Therefore, network analysis, and predicted affinity of bioactives of cocoa towards active site residue of EGFR seems to be in concurrence with these findings and provide possible molecular modes of action of cocoa as a potential anti-cancer nutraceutical. Hirsutrin (galactoside of quercetin) showed stable complex formation with EGFR during the MD simulation. Similarly, hyperosid formed three hydrogen bond interactions and five non-hydrogen bond interactions, in which five interactions were with active site residues. A previous study by Kern et al [36] identified hirsutrin(7.5 μM) and hyperoside (6.7 μM) from apple juice as EGFR-inhibitors. Further, we compared the obtained data concerning a known EGFR inhibitor *i*.*e*. erlotinib, which scored binding energy of—7.0 kcal/mol and formed two hydrogen bond interactions with active site residues.

In the network, arachidonic acid metabolism pathway, cAMP, Rap1, Ras, Phospholipase D, cGMP-PKG, MAPK, PI3K-Akt, and VEGF signaling pathways were found to be the highly enriched pathway modulating multiple protein molecules. On looking into the arachidonic acid metabolism pathway, it includes 7 potential targets i.e. AKR1C3, ALOX12, ALOX5, CYP2C9, PLA2G10, PLA2G2A, and PLA2G5. A previous study demonstrated flavonoids from cocoa to exhibit potent anti-inflammatory effects through modulation of the arachidonic acid pathway by the inhibiting 5-lipoxygenase enzyme [37]. The current study identified flavonoids to disrupt the arachidonic acid pathway by targeting these 7 key protein targets, among which 5-lipoxygenase (ALOX5) and 12-lipoxygenase (ALOX12) are also present. Arachidonic acid metabolism pathway is an important metabolic pathway in which cytochrome P450 (CYP) monooxygenases, cyclooxygenases, lipoxygenases, and phospholipase A2 are potentially involved and play crucial roles in various pathophysiological functions *via* inflammatory response, oxidation, cell proliferation, survival, angiogenesis, invasion, and metastasis, that can promote carcinogenesis [38]. Further, it is well reported from *in vitro* and *in vivo* studies, that flavonoids present in cocoa may act as anti-proliferative, induce apoptosis, and inhibit angiogenesis [39–42]. Also, bioactives present in cocoa *via* catechin, epicatechin, quercetin, and procyanidin, dimer extracts of procyanidin derivatives are reported to down-regulate NF-κB and AP-1 in cancer cell lines [43–46]. Hence, the current study findings on the anti-cancer activity of cocoa could be due to the modulation of the arachidonic acid metabolism pathway and other intracellular signaling pathways *via* cAMP, Rap1, Ras, phospholipase D, cGMP-PKG, MAPK, PI3K-Akt, and VEGF.

The level of reactive oxygen species (ROS) generation in the tissue depends on the balance between oxidants and antioxidants. In endothelial cells, ROS-mediated angiogenesis via various stimuli *via* angiopoietin-I, angiogenin, VEGF, EGF, urotensin-II, shear stress, and hypoxia is a major contribution to cancer [46,47]. The common mechanism involved in cancer is dysregulation of the EGFR that plays a vital role in cell survival, growth, differentiation, and tumorigenesis [48]. Further, angiogenin and VEGF are probably the most widely found initiators of angiogenesis [47,49] and ultimately plays role in the progression of cancer. VEGF activates Rac1- dependent NOX to induce ROS production, which sequentially provokes signaling pathways involved in endothelial cell proliferation and migration and anti-apoptotic cascade [50,51]. In this context, our study further aimed to investigate the *in vitro* antioxidant and cytotoxic potential of the COE. The main goal of cancer chemotherapy is to target cancer cells without exhibiting toxicity to normal cells and this is a limitation of the use of current chemotherapy agents [52].

Therefore, the lead molecule’s antioxidant capacity and selective toxicity on normal and cancer cells must be put into consideration in cancer treatment. Also, there is a close relationship between oxidative stress and the spread of cancer. Several *in vivo* and *in vitro* studies have shown that the use of exogenous antioxidants can prevent free radicals and damage to DNA and proteins, thereby reducing the risk of cancer [53]. The prospect of using natural antioxidants alone or in combination with existing chemotherapy is an ideal strategy to combat tumor development. In this study, DPPH and nitric oxide scavenging activity showed the antioxidant potential of cocoa extract and was found to be equivalent to, ascorbic acid and gallic acid, taken as standards. Further, the MTT cytotoxicity assay demonstrated higher toxicity of COE to EAC compared to CHO. Herein, in the EAC cell line, a well-established cancer cell line with overexpression of EGFR is known to be involved in oxidative stress and cancer progression [17,54,55]. In contrast, the CHO cell line is a normal epithelial cell line that does not express the EGFR [56]. In cancer pathogenesis, rapid activation of the ROS system has been reported [57], which can be neutralized by selected traditional drugs. In this study, we found that COE had a stronger cytotoxic effect on EAC [56] cells compared to CHO. This means that the phytoconstituents contained in bioactives can have a stronger tendency to inhibit the growth of tumor cells than normal cells. As the IC_50_ values of COE were relatively lower in normal cell lines than tumors; may represent some photo components that have a higher binding affinity or modulate proteins/pathways involved in tumor pathogenesis, but not in normal cells [30]. A previous study by Corcuera et al. [58] also demonstrated polyphenols extracted from cocoa as a potential antioxidant agent in HepG2 cells treated with mycotoxins ochratoxin A. Martin *et al*. reported antiapoptotic activity of cocoa polyphenols in tert-butyl hydroperoxide-induced cellular death and apoptosis in HepG2 cells [59]. This antiapoptotic impact was linked to decreased ROS production, avoidance of ERK deactivation and JNK activation, and prevention of caspase-3 activation. Also, numerous researchers demonstrated polyphenolic compounds as potent EGFR tyrosine kinase inhibitors [36,60,61]. Hence, the current study corroborates the previous literature for cocoa bioactives may have more affinity towards cancer cells to inhibit the EGFR task, which was demonstrated *via* docking studies. Further, the antioxidant activity of COE and a pure compound “Hirsutrin” was evaluated for H_2_O_2_ induced oxidative stress in CHO and EAC cell lines. The results showed a protective role of COE and hirsutrin in both the cell lines, in which COE was found to be more potent than hirsutrin. This demonstrates the multiple compounds present in the COE (Fig 10) may pose synergistic activity in the prevention of oxidative stress.

Our study further provides the add-on molecular and bioinformatics support to previous studies by deciphering the possible roles of action of bioactives from cocoa at the molecular level. The entire set of bioactives and study of all of them together along with their interactions may not be feasible although a modest attempt has been made to draw a network to understand the systemic functions to the extent possible within the scope of this study. Our study has used ‘Herbal informatics’ a combination of knowledge on the use of botanicals in traditional medicine with modern-day bioinformatics using computational advancements which is essentially the use of high-tech computational studies and simulations in establishing the validity of existing traditional use through the reverse pharmacology approach. Apart from providing a valuable clue on designing further wet-lab studies on the anti-cancer activity of the COE and hirsutrin our study should help promote the use of cocoa for anti-oxidant and anti-cancer activities and if corroborated which will not only help to validate a traditional medicinal food but is also likely to impact the marketing of cocoa and its products as nutraceuticals. The present study was limited to the use of modern-day informatics and molecular modeling and except for the modest *in vitro* assays; is devoid of biological experimental proof. However, the present study provides vital clues to likely modes of action which could help in designing further research on cocoa.

## Conclusion

The current study used an *in silico* approach followed by an experimental evaluation to investigate the antioxidant and anticancer activity of the cocoa hydroalcoholic extract. We reported interactions of the bioactive from the cocoa with a protein involved in the pathogenesis of cancer which was identified by modulation of multiple pathogenesis/ proteins involved in the ROS system and cancer pathogenesis. Gene set enrichment analysis identified the Arachidonic acid pathway as a likely major target of the bioactives of cocoa to counteract cancer and oxidative stress. Hirsutrin, hyperosid, and other key constituents present in cocoa were found to target and bind with EGFR suggesting their probable roles in inhibiting EGFR and other key protein targets in cancer biology. Further, antioxidant activity validation by *in vitro* study *via* the quantification of enzymatic and non-enzymatic assays and the mechanisms underlying the anti-cancer effect of cocoa extracts/enriched fractions in EAC- induced cancer in mice remain elusive and yet to be evaluated. In addition, as a perspective, the majorly triggered proteins/ targets traced in the network pharmacology need to be further evaluated.

## Supporting information

S1 Table List of phytcompounds selected from *T*. *cocoa* = 54. (XLSX)

S2 Table Probable protein targets of selected phycompounds. (XLSX)

S3 Table Molecular pathways enrichment analysis of probable protein targets. (XLSX)

S1 Graphical abstract (TIF)

## Abbreviations

BE: Binding energy
CHO: Chinese hamster ovary
CID: Compound identification number
CYP: Cytochromes P450
DLS: Drug-likeness score
DOPE: Discrete optimized protein energy
DPPH: 2,2-diphenyl-1-picrylhydrazyl
EAC: Ehrlich ascites carcinoma
EGFR: Epidermal growth factor receptor
FDR: False discovery rate
IC_50_: Inhibitory concentration
KEGG: Kyoto encyclopedia of genes and genomes
NO: Nitric oxide
PDB: Protein data bank
RMSD: Root mean square deviation
RMSF: Root-mean-square fluctuation
ROS: Reactive oxygen species
SMILES: Simplified molecular-input line-entry system
SNP: Sodium nitroprusside
STRING: Search tool for the retrieval of interacting genes/proteins
