# Supplementary figures and images for: Network pharmacology and in vitro testing of Theobroma cacao extract’s antioxidative activity and its effects on cancer cell survival
Source: PLoS One. 2022 Apr 14;17(4):e0259757. doi: 10.1371/journal.pone.0259757 (PMC9009696; doi:10.1371/journal.pone.0259757)

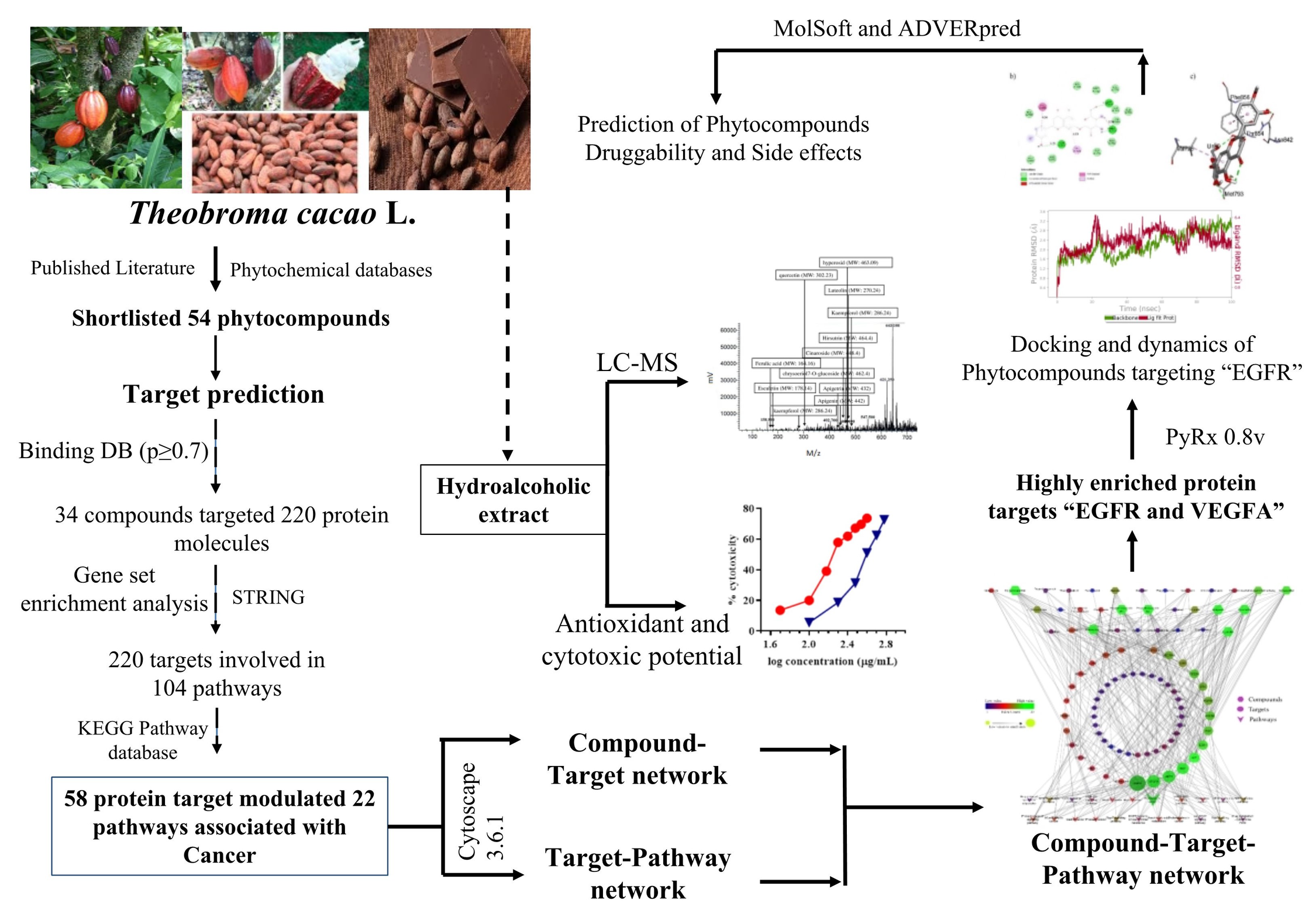

Supplement: S1 Graphical abstract — (TIF) [file pone.0259757.s004.tif]
